# Premature Decline

**Published:** 1859-02

**Authors:** 


### PREMATURE DECLINE.

Many years ago, in travelling among the blue mountains of the Old Dominion, on a visit of curiosity to her "springs," we chanced to fall in with a young clergyman just married. He unfolded to us his prospects, bright and sad—bright as to position and opportunity—sad as to the poor health, which threatened to blast them all. Since then he has risen, and made a high mark among his fellow-men—a mark as good as it is great. A quarter of a century has passed, during which we have never forgotten him, and have never met him; but to-day we received the following:

"Dear Sir—Very highly estimating the ability and utility, the wholesome moral and religious, as well as healthful tenor of your Journal of Health, you will please mail it to me."

He has forgotten that we ever met; but the point of observance is this—the writing is in a hand so trembling, and indicating such bodily debility, that it struck us with amazement. Men of eighty years have written to us in a firmer, bolder, younger hand; and yet he cannot be far from either side of the line of half a century. What changes has time wrought, and how different our constitutions! We are as merry as a cricket and as blithe as a lark of a spring morning in spite of the rubs we have had on land and sea, in city, prairie, or boundless forests of the malarial South. A knowledge and practice of the laws of life unfolds the mystery. He is young enough to electrify the Southern pulpit with his profound and burning eloquence for a quarter of a century to come. But he will never do it, nor for a decade even. Moral:—Theological students ought to spend less time in chewing Hebrew roots and poring over Greek themes—less time in handling theological polemics, and more in studying how to live long, work hard, thrive upon it, and die victorious—the battle won over sin, Satan, and a wicked race.

Let the church in general, and theological professors in particular, remember that a sick soldier is bad enough—he is but a unit—but a sick leader modifies the efficiency of whole regiments. The remedy is patent—let the friends of a sound Christianity look to it.

